# The Asylum Workers' Association: Speeches at the Annual Meeting

**Published:** 1914-05-23

**Authors:** 


### The Asylum Workers' Association.

### SPEECHES AT THE ANNUAL MEETING.

The annual general meeting of the Asylum Workers' Association was held on Wednesday last at the rooms of the Medical Society of London, Sir John Jardine, M.P., occupying the chair.

### The Truth about the Membership.

The annual report and statement of accounts was presented by Sir John Jardine, who commented on the continued decline in the membership of the Association, the aggregate falling-off for the year being 538, and the present membership 3,772. The causes, he said, were well known, but the fact was none the less deplorable, for the greater the numerical strength of the Association the greater the influence which would attend upon its recommendations. The passing into law of the measure dealing with mental deficiency made it incumbent upon the Association to alter its rules so as to admit to its membership workers under the new system. It was important that such workers should have all the protection of an Association such as theirs, which had, they might almost say, its representatives in Parliament. After referring to the losses sustained in the deaths of Sir John Batty Tuke and Miss Honnor Morten, Sir John proceeded to deal more explicitly with the subject of legislation. The Bill to amend the Asylum Officers' Superannuation Act as regards earlier retirement on pension had not made much progress, but it was not going to be dropped. Mr. Duncan Millar and himself had been sympathetically received by the Home Secretary, and but for the extreme pressure of work which had fallen to Mr. McKenna's lot during the last few days, he (Sir John) would probably have had some definite message to bring them that day from the Home Office. They were aspiring to have the help of the Government for their measure, as, without such help, its chances in the present condition of Parliamentary business would be small. He concluded by expressing his indebtedness to Dr. Shuttleworth, the Honorary Secretary, to Dr. James Nicoll, and others, for their great assistance in the course of the year's business, and by moving the adoption of the report.

### The "Ignorance" of Newcomers.

Dr. Hubert Bond seconded the adoption of the report, and expressed the view that the anxieties with which the Association happened to be faced would prove to be temporary and evanescent. The decrease in membership was largely due to the fact that newcomers on the staffs of mental hospitals were not aware of what the Association had done to improve the status of the asylum worker. The Association had been successful in procuring legislation which would be for all time an inestimable boon to mental hospital staffs. The passage of that legislation had made all the difference so far as the future of the asylum worker was concerned. It had removed the element of uncertainty, and he was surprised that there was not a wider recognition of the Association's services in this respect. It was true that prior to that legislation much work was done by two other Associations, but the spade-work of the past was brought to a practical issue by their own body.

### The Necessity of the Association.

There was another direction in which the Association was of value. The relation between doctor and patient, and between nurse, whether male or female, and patient, could never be one of mere contract in pounds, shillings, and pence. It was unthinkable that members of staffs joining the junior ranks of the mental hospital service could become properly trained nurses, male or female, except by the training of the medical men who were colleagues with them in these institutions, and this community of interest pointed to the extreme advantage, and indeed the necessity, for such an Association as theirs.

Mr. Morgans supported the adoption of the report, and said that during the last two or three years there had been a good deal of opposition to the Association, but they were determined to persevere, believing that old-fashioned ways were the best. He agreed with Dr. Bond as to the amount of good work which was done by medical men in asylums, and certainly a great debt was owing to the asylum doctors for the way in which they had endeavoured to improve the conditions of the attendants and nurses.

The adoption of the report was agreed to unanimously.

### The Need for the Present Bill.

Mr. Duncan Millar, M.P., moved the re-election of Sir John Jardine as President, and paid a tribute to Sir John's services on behalf of asylum workers. He had been with Sir John in the Home Secretary's private room, and if the persuasive eloquence of their President, coupled with his pertinacity, could not gain their legislative ends, he did not know what could. He urged the Association to try and get a measure this year which would embody some part of their demands, rather than wait a longer period to secure a larger measure. The Bill now standing in Sir John Jardine's name did secure very substantial advantages, particularly in allowing to women officers a pension irrespective of age (though not of term of service). He hoped that they would be able to adjust the Bill as a Government measure and see it placed on the Statute Book. The Home Secretary was quite alive to the urgent need for something being done to amend the old Asylum Officers Act.

### Enlarging the Association's Scope.

The Mental Deficiency Act, so far as England was concerned, seemed to represent an opportunity for enlarging the scope and activities of the Association. The Scotch measure amended the lunacy law as well as dealt with mental deficiency, and a provision had been incorporated for reckoning time previously served in a parochial asylum, or in the mental ward of a poor-house, to the credit of an established officer or servant in an asylum in Scotland.

### The Claim of Tradesmen Attendants.

Referring to the tradesmen attendants, of whom there were a number attached to a mental hospital in his own constituency, he said that many of them felt the strain of their work very keenly, and the General Board of Lunacy for Scotland was now prepared to recognise the claim under certain conditions for the tradesmen attendants to be included in Class I. In England they were more advanced in this respect than hitherto in Scotland.

Dr. Finegan seconded Sir John Jardine's re-election, and the motion was carried with acclamation. In responding, Sir John Jardine urged that interviews should be sought with, and letters written to, members of Parliament who had mental hospitals in their constituencies, with the object of enlisting their support for the proposed amending legislation.

The election of vice-presidents, central executive committee, and executive officers (there being only a few alterations from the previous year's list) was moved formally by Dr. Rees Philipps, and seconded by Mr. W. A. Jarman, both proposer and seconder expressing the gratitude of the Association for the work of its officers.

An Alteration of the Rules.

The names as submitted were unanimously accepted by the meeting, and then the Rev. John Peck moved, and Dr. Shuttleworth seconded, certain verbal alterations in the Association's rules, with the object of including amongst the persons eligible for membership workers and others interested in the care of the mentally defective under the provisions of the Mental Deficiency Acts of 1913. This motion also was carried unanimously.

Dr. Bower moved a vote of thanks to the President and speakers, and Dr. Seward seconded, the latter remarking that in connection with the Mental Deficiency Act it was probable the ultimate result would be to make the lot of the ordinary asylum worker somewhat harder than before. Many of the quieter patients would be removed to other institutions under the new Act, and it would mean that mental hospital workers would be left to deal with the more acute and difficult cases.

